# Leiomyosarcoma of the Vagina: An Exceedingly Rare Diagnosis

**DOI:** 10.1155/2015/363895

**Published:** 2015-12-09

**Authors:** Nathan A. Keller, Heidi Godoy

**Affiliations:** ^1^Department of Obstetrics and Gynecology, Albany Medical Center, 16 New Scotland Avenue, Second Floor, MC-74, Albany, NY 12208, USA; ^2^Women's Cancer Care Associates, 319 South Manning Boulevard, Suite 301, Albany, NY 12208, USA

## Abstract

*Background*. Primary leiomyosarcoma of the vagina is an exceedingly rare diagnosis. Current estimates are that this tumor could at most represent a mere 0.062% of malignant neoplasms in the female genital tract, although in actuality it is likely far less common.* Case Presentation*. A 70-year-old female gravida 3 para 2 with new onset palpable vaginal mass and pink vaginal discharge is diagnosed with primary leiomyosarcoma of the vagina. Chemotherapy is complicated by acute Lyme disease, and the patient requires a robotic-assisted total hysterectomy with bilateral salpingo-oophorectomy and partial vaginectomy. The patient remains without recurrence 18 months after surgery.* Conclusion*. Vaginal leiomyosarcoma is exceedingly rare with an aggressive course, high recurrence, and undetermined ideal treatment regimen. Its diagnosis can be delayed and its presentation varied. Information on this rare tumor type is predominantly through rare case reports with collective consensus on management lacking. The gynecologic oncologist must exercise prudence in individualizing treatment regimens for this rare yet aggressive malignancy.

## 1. Introduction

Primary leiomyosarcoma of the vagina is exceedingly rare. The preponderance of carcinomas of the vagina are secondary, with primary tumors representing a much smaller entity [1]. For instance, about 84% of carcinomas involving the vagina are secondary [2]. Of these secondary tumors, about 32% are from the cervix, 18% from the endometrium, 9% from the colon and rectum, 6% from the ovary, and 6% from the vagina [2]. Primary vaginal carcinomas represent 2-3% of malignant neoplasms in the female genital tract [1]. Of the various histological types of primary vaginal tumors, squamous cell carcinomas are by far the most common type comprising about 82.6% of primary tumors [1]. Alternatively, primary vaginal sarcomas account for only 3.1% [1]. Of the primary vaginal sarcomas, primary vaginal leiomyosarcomas again represent only a fraction. This means that if every primary vaginal sarcoma was a primary vaginal leiomyosarcoma, primary vaginal leiomyosarcoma would represent a mere 0.062% of malignant neoplasms in the female genital tract. In actuality, since this is not the case, the incidence is likely yet far smaller. In fact, literature reviews in the year 2000 found fewer than 70 cases reported in English literature [3]. This case report represents one such case. The incidence, diagnosis, and treatment of primary leiomyosarcoma of the vagina will be reviewed in this case report. Hopefully this case report will add further meaningful insight into this rare malignancy.

## 2. Case Presentation

A 70-year-old gravida 3 para 2 presented with a palpable vaginal mass and complaints of pink vaginal discharge and perineal discomfort when she sat down over the previous three months. Her past medical history was significant for morbid obesity, arthritis, fibromyalgia, hypertension, depression, and anxiety. She had two previous full term vaginal deliveries and one spontaneous miscarriage and underwent menopause at age 51. Family history was significant for a maternal grandmother, paternal grandmother, and maternal aunt with breast cancer all diagnosed in their seventies and eighties. She denied tobacco, alcohol, or illicit drug use and was retired.

On physical examination there was a palpable mass in the posterior lower one-third of the vagina. Transvaginal ultrasound demonstrated 3.0 × 2.5 × 2.2 cm, 1.8 × 1.7 × 1.7 cm, and 0.8 × 0.6 × 0.8 cm vascular lesions within the lower posterior vaginal wall. CT scan of the chest did not demonstrate any evidence of metastatic disease and MRI of the abdomen and pelvis demonstrated only a uterine fibroid, which was not concerning for malignancy. Given the unknown origin of the 3 vaginal masses, resection in the operating room was performed.

Two specimens were sent to pathology both showing a smooth muscle neoplasm with increased mitotic activity and moderate to severe cytologic atypia, consistent with leiomyosarcoma. Sections revealed spindle cell neoplasm with moderate to severe cytologic atypia and increased mitotic activity (focally up to 8 per ten high power fields) with focal coagulative necrosis. Immunohistochemical stains were positive for vimentin, desmin, and SMA in tumor cells and were negative for S100, Melan-A, myogenin, and cytokeratin AE1/3. An immunohistochemical stain for Ki-67 was positive and suggested an increased proliferative index (10–15%).

According to current classification criteria, it is recommended that smooth muscle tumors of the vagina that are greater than 3.0 cm in diameter (both specimens #1 and #2 measure 3.5 cm in aggregate), with five or more mitotic figures per ten high power fields (present in both specimens #1 and #2), and with infiltrating margins be classified as leiomyosarcoma [4]. Margins were positive in both specimens. Pelvic washings were negative for malignant cells. Surgical resection in sum showed presence of disease in the vagina but not elsewhere, making the final diagnosis leiomyosarcoma of the vagina an exceedingly rare diagnosis.

The patient was discussed at local tumor board and a regimen of chemotherapy with gemcitabine/docetaxel ×4 cycles with plans for surgery afterward was decided upon. During gemcitabine/docetaxel therapy the patient's course was complicated by acute Lyme disease with a visible target lesion on the patient's right arm. She was consequently treated with doxycycline in the midst of her first cycle. After her third cycle the patient had severe fatigue and left lower extremity cellulitis. She was hospitalized for 5 days in the setting of pancytopenia and given IV antibiotics. The 4th cycle of gemcitabine/docetaxel was not given per patient request as she felt unable to tolerate the regimen. Consequently, plans were made to take the patient to the OR instead of receiving additional chemotherapy. She underwent a robotic-assisted hysterectomy, bilateral salpingo-oophorectomy, cystoscopy, and resection of residual vaginal tumor. Surgical pathology showed spindle cell neoplasia from the vaginal tissue consistent with the previous diagnosis of leiomyosarcoma of the vagina. Her uterus, cervix, fallopian tubes, and ovaries showed leiomyomas, but no malignancy. Her pelvic washings were negative. The patient was then treated with standard field pelvic radiation with vaginal high dose radiation for a total dose of 6600 cGy. The patient has remained cancer-free to date, 18 months after surgery.

## 3. Discussion

Leiomyosarcomas are largely considered to be a uterine neoplasm and typically arise de novo from uterine smooth muscle, with a rare few originating from preexisting leiomyomas. Despite this rare transformation from a leiomyoma to a leiomyosarcoma, it is not uncommon to find both leiomyomas and leiomyosarcomas in the same specimen. For instance, approximately 0.5% of women who have hysterectomies for leiomyomas are found to also have leiomyosarcomas [5]. The differentiation of the two can only be made histologically where leiomyosarcomas contain marked atypia, from highly differentiated cells to highly pleomorphic and anaplastic cells [6]. Nuclear atypia, mitotic index, and zonal necrosis are parameters used in the differentiation from leiomyomas [6]. It is generally accepted that the presence of 10 or more mitoses per 10 high power fields indicates malignancy [6]. Cytological atypia and necrosis further bolster the diagnosis [6]. Further, if either nuclear atypia or epithelioid cells are present, 5 mitoses per 10 high power fields are sufficient for the diagnosis of leiomyosarcoma [6]. Unlike leiomyomas, leiomyosarcomas have complex and often variable karyotypes [6].

When arising from the uterus, these smooth muscle tumors are most commonly seen between the ages of 45 and 55 and can be quite devastating with potential early metastases often by hematogenous spread [1, 7]. These tumors are known for their very aggressive nature and poor prognosis likely secondary to hematogenous metastasis. For instance, one study of autopsies showed that metastases to distant locations such as the lungs often occurred without lymphatic disease, supporting a hematogenous mode of dissemination [8]. In fact more than half eventually metastasize hematogenously to distant organs. Leiomyosarcomas also have racial variations with African American women having twice the incidence of leiomyosarcoma compared to Caucasian women [9].

Leiomyosarcomas of the vulva and vagina are much rarer entities. For instance, if primary vaginal carcinomas represent 2-3% of malignant neoplasms in the female genital tract, with primary vaginal sarcomas accounting for about 3.1% of these, primary vaginal sarcomas would represent a mere 0.062% of malignant neoplasms in the female genital tract [1]. Since primary leiomyosarcomas represent only a fraction of primary vaginal sarcomas, primary leiomyosarcomas likely represent much less than 0.062% and are thus exceedingly rare. The presentation of this malignancy can vary, but generally patients with this malignancy will present with an asymptomatic vaginal mass [10]. In other cases, such as in our case, vaginal discharge or bleeding may also be present. Vaginal leiomyosarcomas may originate in any part of the vagina but are predominantly submucosal [10]. Complicating matters further, when the malignancy occurs in more distal parts of the vagina or vulva a resemblance to Bartholin duct cysts can cause a delay in diagnosis [11].

There is little literature available on vaginal sarcomas, most of which are case reports [10]. For instance, a recent review in India found that only about 71 cases have ever been reported in the English literature [10]. This paucity of literature makes ideal management of these tumors difficult, and most gynecologic oncologists will go through their whole career without seeing a single case. As such, the optimal treatment modalities have not been established. Primary treatment typically consists of resection of the tumor with adequate margins [10]. After resection of the tumor, the ideal management is less well defined. For example, one study noted that only patients treated with pelvic exenteration had long-term survival [12], but given the radical nature of this procedure patients should be selected carefully. In a review performed by Ciaravino and colleagues, survival probabilities were calculated using the Kaplan-Meier method, and the overall probability of 5-year survival was found to be 43% [3]. This same study indicated that age directly affected prognosis as patients over 50 had a five-year survival of 26%, while those under 40 had a five-year survival of 51% [3]. Not surprisingly, these authors also found that 5-year survival was directly influenced by stage, with 55% and 44% for stages I and II, respectively, and 25% survival at 18 months for a combined stage III/IV group [3].

While surgery is considered the mainstay of treatment, this study also showed that there was no difference in survival between patients who had surgery alone and those who had surgery followed by adjuvant radiotherapy or chemotherapy [3]. Alternatively, no patient treated primarily with chemotherapy or radiation therapy survived past 2 years [3]. However, those treated primarily with surgery had a survival rate of 57% at 5 years [3]. As such, the respective roles of chemotherapy and radiotherapy in leiomyosarcoma are not adequately defined at this time [3]. Radiotherapy may best be used to control local disease [3]. The available literature strongly indicates that primary surgical management continues to be the most viable option.

This case illustrates an example of how a multimodality treatment regimen resulting in a cancer-free patient 18 months after surgery can be utilized in the treatment of vaginal leiomyosarcoma. Specifically, in our case, chemotherapy was initiated after the initial resection of the vaginal leiomyosarcoma was complete. When pathology showed that the tumor was a leiomyosarcoma, chemotherapy had already been begun. Given the rarity of vaginal leiomyosarcoma, it was thought that this was of a likely uterine source and consequently at that time a resection of residual vaginal tumor, a robotic-assisted hysterectomy, and bilateral salpingo-oophorectomy with cystoscopy were performed. After this was complete, standard field pelvic radiation with vaginal high dose radiation was performed. This multimodality method of treatment in our case appears to be highly effective to date, suggesting a potential role for both chemotherapy and radiotherapy in treatment of leiomyosarcoma of the vagina. Interestingly, a similar case of leiomyosarcoma of the vagina, in which hysterectomy, upper vaginectomy, and pelvic radiation alone were utilized without initial chemotherapy, resulted in pulmonary recurrences after merely three months [13]. Only then was chemotherapy utilized in this study [13]. Our early adjuvant use of chemotherapy may reduce such recurrences.

In conclusion, vaginal leiomyosarcoma is an exceedingly rare diagnosis with an aggressive course, high recurrence, and undetermined ideal treatment regimen. Its diagnosis can be delayed and its presentation varied. Information on this rare tumor type is predominantly through rare case reports, and collective consensus on ideal management is lacking. As such, the gynecologic oncologist must exercise prudence in individualizing treatment regimens for this rare, yet aggressive, malignancy.

## References

[1] Berek J. S., Hacker N. (2010). *Gynecologic Oncology*.

[2] Fu Y. S. (2002). *Pathology of the Uterine Cervix, Vagina, and Vulva*.

[3] Ciaravino G., Kapp D. S., Vela A. M. (2000). Primary leiomyosarcoma of the vagina: a case report and literature review. *International Journal of Gynecological Cancer*.

[4] Kurman R. J., Ellenson L. H., Ronnett B. M. (2011). *Blaustein's Pathology of the Female Genital Tract*.

[5] Harry V. N., Narayansingh G. V., Parkin D. E. (2007). Uterine leiomyosarcomas: a review of the diagnostic and therapeutic pitfalls. *The Obstetrician & Gynaecologist*.

[6] Kumar V., Abbas A. K., Fausto N., Aster J. C. (2010). *Robbins and Cotran Pathologic Basis of Disease*.

[7] Gockley A. A., Rauh-Hain J. A., Del Carmen M. G. (2014). Uterine leiomyosarcoma: a review article. *International Journal of Gynecological Cancer*.

[8] Rose P. G., Piver M. S., Tsukada Y., Lau T. (1989). Patterns of metastasis in uterine sarcoma. An autopsy study. *Cancer*.

[9] Toro J. R., Travis L. B., Wu H. J., Zhu K., Fletcher C. D. M., Devesa S. S. (2006). Incidence patterns of soft tissue sarcomas, regardless of primary site, in the surveillance, epidemiology and end results program, 1978–2001: an analysis of 26,758 cases. *International Journal of Cancer*.

[10] Khosla D., Patel F. D., Kumar R. (2014). Leiomyosarcoma of the vagina: a rare entity with comprehensive review of the literature. *International Journal of Applied and Basic Medical Research*.

[11] González-Bugatto F., Añón-Requena M. J., López-Guerrero M. A., Báez-Perea J. M., Bartha J. L., Hervías-Vivancos B. (2009). Vulvar leiomyosarcoma in Bartholin's gland area: a case report and literature review. *Archives of Gynecology and Obstetrics*.

[12] Peters W. A., Kumar N. B., Andersen W. A., Morley G. W. (1985). Primary sarcoma of the adult vagina: a clinicopathologic study. *Obstetrics and Gynecology*.

[13] Anderson M. L., Bodurka D. C. (2008). Thoracotomy for the management of recurrent vaginal leiomyosarcoma. *International Journal of Gynecological Cancer*.

